# Modelling the Progression of Male Swimmers’ Performances through Adolescence

**DOI:** 10.3390/sports4010002

**Published:** 2016-01-14

**Authors:** Shilo J. Dormehl, Samuel J. Robertson, Craig A. Williams

**Affiliations:** 1 University of Exeter, Children’s Health and Exercise Research Centre, College of Life and Environmental Sciences, St Luke’s Campus, Heavitree Road, Exeter EX1 2LU, UK; sjd223@exeter.ac.uk; 2 Victoria University, Institute of Sport, Exercise & Active Living, Melbourne 3011, Australia; Sam.Robertson@vu.edu.au

**Keywords:** adolescent, specialisation, quadratic functions, talent-identification, sub-elite

## Abstract

Insufficient data on adolescent athletes is contributing to the challenges facing youth athletic development and accurate talent identification. The purpose of this study was to model the progression of male sub-elite swimmers’ performances during adolescence. The performances of 446 males (12–19 year olds) competing in seven individual events (50, 100, 200 m freestyle, 100 m backstroke, breaststroke, butterfly, 200 m individual medley) over an eight-year period at an annual international schools swimming championship, run under FINA regulations were collected. Quadratic functions for each event were determined using mixed linear models. Thresholds of peak performance were achieved between the ages of 18.5 ± 0.1 (50 m freestyle and 200 m individual medley) and 19.8 ± 0.1 (100 m butterfly) years. The slowest rate of improvement was observed in the 200 m individual medley (20.7%) and the highest in the 100 m butterfly (26.2%). Butterfly does however appear to be one of the last strokes in which males specialise. The models may be useful as talent identification tools, as they predict the age at which an average sub-elite swimmer could potentially peak. The expected rate of improvement could serve as a tool in which to monitor and evaluate benchmarks.

## 1. Introduction

Elite-level athletes have been well characterised compared with sub-elite adolescents. Retrospective studies such as those conducted by Sokolovas [1], Costa *et al.* [2] and Allen *et al.* [3] have enabled the performance of individual top-ranked swimmers to be tracked as they progressed through their careers, in the anticipation that the process may map the path to potential elite performance. The majority of longitudinal studies in swimming have also focused on adult athletes who had already reached elite status. These studies aimed to characterise both the consistency and rate of development of individual performances [2,3,4,5] *inter alia* and claimed to enable practitioners the ability to predict potential medallists and/or determine realistic individual performance goals [6,7].

Talent identification (TI) has been defined as the process whereby current performers are recognised as having the potential to become future elite athletes and talent development (TD) as the provision of a suitable, opportunity-rich, learning environment [8]. As improved TI and TD programmes become more widely implemented, former and current elite athletes are unlikely to reflect the pathway of future champions because they were products of an era of rudimentary TI practices. Furthermore it is likely that there would be a decrease in the mean age to reach elite-level standard and the start of the “peak-performance window”, a term coined by Allen, Vandenbogaerde and Hopkins [3]. Therefore current “atypically-young” elite athletes could become the future norm.

To date, the prediction of future talent in young children has seldom shown to be accurate [9,10]. The majority of TI programmes continue to be based on cross-sectional studies [9] despite it being evident that many of the physical components being assessed may change or may not be as important at the elite adult level [11]. If future talent is to be successfully identified in adolescents, sporting organisations should aim to avoid summative discrete measurements and rather focus on creating environments conducive to longitudinal formative assessments [12]. This is pertinent because skill acquisition is non-linear and athletes and their training environments should thus be considered as complex dynamic systems [13].

However, since there is a dearth of longitudinal research on athletes of this age, there is a need to better understand the broad pool from which future talent is likely to arise, *viz*. the sub-elite athlete. Sub-elite athletes have been defined as those athletes who are yet to represent their country at international level [14]. Despite the challenges of categorising such an unstable, developing group of athletes, it is important to prioritise this often overlooked category of swimmer if the use of valid performance measures in TI is to progress.

The consequences of poor TI processes result in many late-developing athletes with potential being de-selected, frequently leading to dropout and often result in exclusivity rather than inclusivity [15]. Another outcome of many dated TI processes is that they inevitably lead to athletes choosing to specialise early in a sport in which they are believed to show potential. This practice may be due to the fact that immediate successful performance is prioritised over unrealised potential in the long term [10].

A further limitation that potentially explains the lack of success of many TI approaches relates to the types of assessments used. Specifically, many TI assessments fail to accurately represent the competition setting, often relying on generic physical evaluations or closed skill tests that do not correlate well with the demands that athletes will face in competition [16]. The rationale for undertaking this longitudinal study of male adolescent swimmers, in the competitive setting, was to: (a) model performance progression through adolescence; and (b) predict the ages at which performance plateaued. Advancements in statistical modelling have recently provided researchers with a more refined and rigorous method of interpreting longitudinal data [17,18], but until now, sub-elite athletes have been underrepresented. Additionally, swimming has commonly been considered as a single sport [3,19], rather than a sport with multiple specialisms in the form of different stroke techniques and distances. Where the individual strokes have been characterised, it is still rare to find simultaneous analyses of all strokes together. The aim of this study was to create a tool that will enable the progression and variability of performances throughout puberty to be tracked in relation to a modelled mean and as such, assist coaches with the setting of realistic goals when devising individually-tailored training programmes. Finally, the models produced in this study could potentially be used as instruments for identifying talented young male swimmers.

## 2. Methods

Performance times for all male entrants (*n* = 446, aged between 12 and 19 years) who competed in one of seven individual events (Table 1) were extracted from the official results of an annual international schools swimming championships from 2006 to 2013. The data was in the public domain and downloaded from the relevant tournament websites. All swimmers from the 13 competing schools were assigned individual identity codes to ensure anonimity. The study was approved by the institutional ethics committee and conformed to the recommendations of the Declaration of Helsinki. The number of observations in each of the seven events entered over the 8 year analysis period are described in Table 1. The swimmers’ ages at the time of each competition were also obtained.

**Table 1:** Cumulative number of performances (between 2006 and 2013) for male swimmers between the ages of 12 and 19 years in each event.

Number of Performances (years)	50 m Freestyle	100 m Freestyle	200 m Freestyle	100 m Backstroke	100 m Breaststroke	100 m Butterfly	200 m Individual Medley
1	376	280	190	178	196	132	139
2	151	103	87	74	69	55	65
3	69	49	37	34	37	26	38
4	25	17	16	14	21	14	18
5	9	3	6	1	9	4	6
6	2	1	1	0	3	2	1

### 2.1. Statisical Analysis

The raw datasets for all performances in each of the seven events were tested for normality using the Shapiro-Francia test in STATA ver. 13. The datasets for all events had non-normal distributions. The trajectories of the curves showing the progression in performance during adolescence were analysed using mixed or multi-level modelling (MLM) in STATA. Time was zero centred at the first point of observation (12 years of age), using an unstructured covariance approach. The fit of the models for fixed and random effects were compared by obtaining maximum likelihoods using a hierarchical method. The final models were quadratic functions for fixed effects (*y* = a*x*^2^ + b*x* + c). The fixed effects of time represented polynomial changes of the population with age and the random effects reflected individual deviations from the sample mean trajectory. Inter-class correlations were calculated and R^2^ values determined in order to measure the difference between and within person variability and effect size respectively.

### 2.2. Evaluation of Models

Cross-validation of models is highly recommended to ensure the generalizability of the findings [20]. Cross-validation was therefore performed for each of the seven models separately, whereby the datasets were randomly split into training (66%) and test (33%) sets. Performance of each test set was determined through obtaining the mean difference in model performance.

The percentage rate of improvement was determined through differentiation of the quadratic functions for each event separately, as $y=\left(\frac{2a}{c}\;\times \;100\right)x+\left(\frac{b}{c}\;\times \;100\right)$, where *y* = % change in performance time and *x* + 12 = age, in years. The age at peak performance was calculated as the axis of symmetry of the quadratic function *i.e.*, $\frac{-b}{2a}$.

## 3. Results

The models for all three freestyle events and the backstroke event resulted in fixed quadratic random linear functions whereas those for the remaining three events showed the best fit as fixed quadratic random intercept functions (Table 2). The high ICC values for all the models indicated a greater variability between- rather than within-swimmers. The model for the means explained 16%–25% of the variance in the changes with age. The results from the cross validation indicated that the fixed effects of the quadratic functions for all events fell within the 95% confidence intervals (C.I.) of those of the full models, with the exception of the 100 and 200 m freestyle events (Table 2). The 1/3 and 2/3 subsamples for both of these events fell marginally outside of the C.I. of the full model for their fixed intercepts only.

**Table 2:** Summary of models for all events with cross validation for each of the fixed effects of the quadratic functions.

Predictor	50 m Freestyle		100 m Freestyle		200 m Freestyle		100 m Backstroke		100 m Breaststroke		100 m Butterfly		200 m Individual Medley	
	Mean	*P*	Mean	*P*	Mean	*P*	Mean	*P*	Mean	*P*	Mean	*P*	Mean	*P*
Fixed Quadratic (a)	0.21	<0.001	0.48	<0.001	0.93	<0.001	0.47	<0.001	0.50	<0.001	0.41	<0.001	0.97	<0.001
Standard error (SE)	(0.02)	–	(0.06)	–	(0.14)	–	(0.08)	–	(0.08)	–	(0.11)	–	(0.14)	–
95% C.I.	0.05	–	0.11	–	0.28	–	0.16	–	0.16	–	0.21	–	0.28	–
Cross val. 2/3 diff.	0.003	<0.001	0.051	<0.001	0.21	<0.001	0.07	<0.001	−0.068	<0.001	0.05	0.007	0.10	<0.001
Cross val. 1/3 diff.	0.03	<0.001	0.010	<0.001	0.003	0.001	−0.05	0.001	0.097	0.005	−0.13	0.001	−0.22	<0.001
Fixed Linear (b)	−2.78	<0.001	−6.38	<0.001	−12.16	<0.001	−6.37	<0.001	−6.65	<0.001	−6.40	<0.001	−12.56	<0.001
(SE)	(0.18)	–	(0.45)	–	(1.11)	–	(0.62)	–	(0.62)	–	(0.85)	–	(1.06)	–
95% C.I.	0.36	–	0.88	–	2.18	–	1.22	–	1.22	–	1.66	–	2.08	–
Cross val. 2/3 diff.	−0.04	<0.001	−0.44	<0.001	−2.01	<0.001	−0.62	<0.001	0.31	<0.001	−0.41	<0.001	−0.94	<0.001
Cross val. 1/3 diff.	−0.16	<0.001	0.28	<0.001	0.37	<0.001	0.23	<0.001	−0.22	<0.001	1.11	<0.001	1.23	<0.001
Fixed Intercept in seconds (c)	37.23	<0.001	83.81	<0.001	179.45	<0.001	95.33	<0.001	104.35	<0.001	92.79	<0.001	195.98	<0.001
(SE)	(0.38)	–	(0.99)	–	(2.39)	–	(1.37)	–	(1.12)	–	(1.65)	–	(2.21)	–
95% C.I.	0.74	–	1.94	–	4.67	–	2.68	–	2.34	–	3.23	–	4.33	–
Cross val. 2/3 diff.	0.07	<0.001	1.48	<0.001	5.36	<0.001	1.57	<0.001	0.59	<0.001	0.77	<0.001	1.86	<0.001
Cross val. 1/3 diff	0.17	<0.001	−2.47	<0.001	−3.97	<0.001	−0.88	<0.001	0.33	<0.001	−2.19	<0.001	−3.26	<0.001
Interclass correlation (ICC)	0.95		0.97		0.96		0.97		0.91		0.90		0.92	
Wald’s χ^2^	543.36 (df = 7)		479.95 (df = 7)		315.318 (df = 7)		298.98 (df = 7)		430.27 (df = 5)		318.57 (df = 5)		461.07 (df = 5)	
Total R^2^	0.25		0.23		0.16		0.17		0.20		0.22		0.17	
*n*	376		280		190		178		196		132		139	

Note: Cross val. diff. is the difference between the cross validation split and the whole sample. Wald’s χ^2^ is Wald’s chi-square.

**Figure 1:**
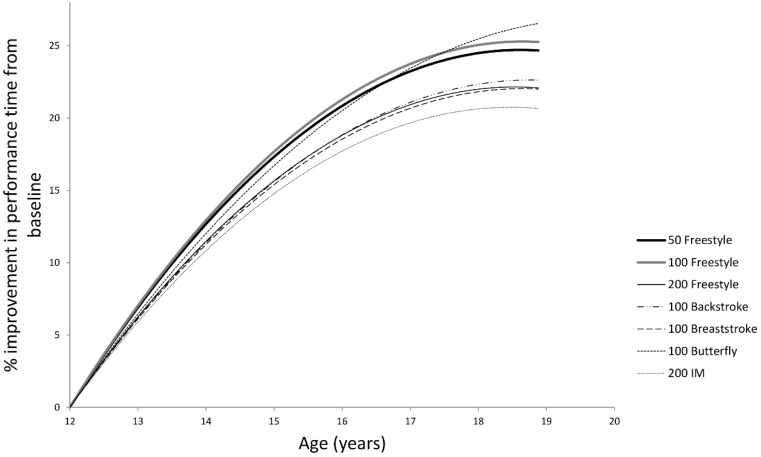
Quadratic functions of the progression in performance for each of the seven events modelled for males from the baseline of 12 years through to 19 years of age.

Similar distinct trends in the trajectories of the models were observed when compared to threshold age of peak performance or a fixed age of 18 years (Figure 1). The progression of swimmers of 100 m butterfly differed from all the other events. The butterfly swimmers reached a peak performance age more than a year later than those in other events, but displayed the highest rate of improvement (Table 3). In contrast, the slowest rate of improvement and joint youngest age of peak performance occurred for swimmers of the 200 m individual medley (IM). Of the remaining events, two groups emerged; 50 and 100 m freestyle events showed very similar trajectories, as did the 100 m backstroke, breaststroke and 200 m freestyle (Figure 1, Table 3).

**Table 3:** Descriptors determined for the full models of the seven events.

Predictor	50 m Freestyle	100 m Freestyle	200 m Freestyle	100 m Backstroke	100 m Breaststroke	100 m Butterfly	200 m Individual Medley
% Rate of improvement (12 year—peak age)	24.70	25.29	22.15	22.64	22.03	26.92	20.75
% Rate of improvement (from 12 to 18.5 year)	24.70	25.28	22.15	22.60	22.02	26.16	20.75
Threshold age in peak performance (year)	18.5 (0.12)	18.7 (0.06)	18.6 (0.14)	18.7 (0.08)	18.6 (0.08)	19.8 (0.11)	18.5 (0.14)
Performance time (s) at threshold age	28.26 (0.22)	62.44 (1.53)	139.54 (9.38)	73.94 (2.07)	82.26 (2.40)	67.93 (3.09)	155.35 (9.36)

Data given as mean with standard errors shown in brackets.

## 4. Discussion

This is the first study to longitudinally assess the development of sub-elite adolescent male swimmers in multiple stroke techniques during competition, where the primary purpose was to generate a TI tool for young male swimmers. To achieve this, the performance progression of these swimmers was modelled to identify the mean threshold age at which they could be expected to start competing at the highest level. It was thus unsurprising that for most events, age correlated with the widely accepted age of biological maturity for males (Table 3) in agreement with previous work [21,22]. Hence it could be surmised that any further gains in performance from this point onwards, would potentially come from the biomechanical and psychological domains. However, the rate of progression in performance of male athletes, including swimmers, during adolescence is known to be attributed predominantly to the tempo of their anthropometric and physiological maturation [23].

There was a narrow range of ages (18.5–19.8 years) at which the swimmers approached their peak in performance in the seven events modelled in this study. In a retrospective study of career progression in elite swimmers, Allen, Vandenbogaerde and Hopkins [3] found ages of peak performance that were on average 6.2 years older than those for the corresponding events in this study. The discrepancy between the two studies can be explained by three factors. Firstly, Allen, Vandenbogaerde and Hopkins [3] tracked athletes back over a longer career path (from the ages of 12–30 years). Secondly, their sample only contained athletes who had already progressed to the elite level (probably through simplistic TI systems), whereas the talent pool in this study contained a wider-ranging standard of swimmers. Thirdly, the majority of their sample would have comprised athletes who grew up prior to the long-term athlete development (LTAD) generation of the post millennium era that our sample contains. But there is in fact no discordance between these two studies; they are complementary. While Allen, Vandenbogaerde and Hopkins [3] identified a potential absolute peak in performance of current and past elite swimmers, this study, based on the broad nature of the sample, provides insight into the threshold age of performance; *i.e.*, the cusp of the window of peak performance for future talented swimmers. The discrepancy between the two predicted ages therefore serves as a period of potential performance development that will come about through factors such as training interventions and increased experience, rather than maturational development. The models in Allen, Vandenbogaerde and Hopkins [3] are therefore more likely to provide coaches with a selection tool for swimmers currently in an elite squad, rather than as a means to identify future talent.

Freestyle is the most efficient stroke [24,25] and is most frequently swum in practice. It is therefore unsurprising that two of the three steepest progressions of performance occurred in freestyle events (Table 3), since this stroke contributes in excess of 30% of the events on offer at the majority of competitions [26,27,28]. A study by Costa, Marinho, Bragada, Silva and Barbosa [2] on elite Portuguese male freestyle swimmers, found similar rates of progression (14.36%–18.97%) between the ages of 12 and 18 years.

Vaso *et al.* [29] hypothesised that IM swimmers might peak later than those in freestyle events, due to the additional skills required in mastering all four of the recognised stroke techniques and their unique turns in this complex event. In contrast, our models suggested that male IM swimmers peaked in performance earlier than the majority of other stroke specialisms (Table 3), but showed the slowest rate of improvement from 12 to 19 years (Figure 1). There are a number of reasons that may explain this observation. Firstly, IM swimmers may have started swimming at a young age, suggesting they may have a more advanced level of biomechanical experience and learning than swimmers in other events. Nevertheless, IM swimmers may not yet have found their specialist stroke. Once they do however, these swimmers may then focus more on other events. Secondly, many LTAD-based programmes advocate that coaches discourage swimmers from specialising too early in one stroke, in favour of focusing on the continued development of all strokes [30].

The butterfly swimmers in this study reached their threshold in peak performance at a later age than all other events (Table 3). They did however demonstrate the greatest rate of improvement. The lag could be related to the need to gain sufficient experience in the stroke in order to efficiently coordinate its propulsive biomechanical actions. The butterfly stroke relies heavily on the precision of timing of its propulsive and recovery phases and consequently when these are not optimised, the rhythm becomes compromised [31]. This means that when these factors do come together, considerable performance improvements are realised.

### 4.1. Limitations

Being a school-level competition, the sample lacks the homogeneity of an exclusive elite-level sample, however cross validation of the models supported their generalizability. This could be seen as a limitation of the study as it does not distinguish between early and late maturers, the former of which would be more likely to have been identified as being talented within this age range [32]. But, the decision to use longitudinal competition data, so as to maximise external validity, came at the cost of collecting any corresponding anthropometric data to assess maturation.

The models fit most events well with the exception of the 100 and 200 m freestyle (Table 2). The youngest swimmers of these events were markedly variable in standard. Dormehl and Williams (2016) [33], did however find a lack of stability in the continued selection of these events by male swimmers between the ages of 12 and 19 years. Freestyle is also likely to be the stroke most favoured by inexperienced competitive swimmers, since it is the most efficient stroke with which they would be most familiar [25].

### 4.2. Practical Applications

Until now, quantifying deviations from average performances in adolescents has been subjective, due to the intertwined relationship between puberty, skill acquisition and underlying talent. The value of these models is that they can be used to determine if adolescent male swimmers were under- or over-performing based on their current age, because they were derived using athletes from a wide range of abilities and different stages of development. Consequently, deviations from the projected performance trajectories could be attributed to a swimmer’s stage of maturational development and/or potentially the standard of coaching received. The models could be useful as a TI tool, as they estimate the age at which an average sub-elite swimmer could potentially peak. Furthermore, the expected rate of improvement would serve as a useful target-setting tool for coaches and the authors propose that this model could serve as a prototype, from which further refinements could be expected to evolve.

## 5. Conclusions

Separating the performance gains as a result of the combined effects of maturation and training interventions remains an elusive goal for sports scientists. This is the first study that has attempted to model the progression of sub-elite adolescent male swimmers’ performances across a multitude of individual strokes and distances. These models have identified that swimmers improve at different rates and achieve peak performances at different ages in different events ranging from 18.5 and 19.6 years of age. Assessing the gap between reaching biological maturity and peak performance should however continue to be an area of interest to the research community. Effectively identifying the potential “trainability” of young but mature athletes could hold the key to further improvements in athletic performance, especially in late specialisation sports, such as swimming.
